# Electron Beam Irradiation Alters the Physicochemical Properties of Chickpea Proteins and the Peptidomic Profile of Its Digest

**DOI:** 10.3390/molecules28166161

**Published:** 2023-08-21

**Authors:** Yaqi Zhang, Yunfei Kong, Wanjun Xu, Zhen Yang, Yulong Bao

**Affiliations:** 1 School of Food and Biological Engineering, Jiangsu University, Zhenjiang 212013, China; zhangyaqi160@163.com (Y.Z.); kyf980206@163.com (Y.K.); x1779961516@163.com (W.X.); 2 Key Laboratory of Nuclear Agricultural Sciences, Ministry of Agriculture and Zhejiang Province, Institute of Nuclear Agricultural Sciences, Zhejiang University, Hangzhou 310058, China; zhen.yang@zju.edu.cn

**Keywords:** chickpea protein concentrate (CPC), electron beam irradiation (EBI), in vitro digestion, peptidomic profile

## Abstract

Irradiation can be used for the preservation of chickpea protein as it can destroy microorganisms, bacteria, virus, or insects that might be present. However, irradiation may provoke oxidative stress, and therefore modify the functionality and nutritional value of chickpea protein. In order to study the effects of irradiation on the physicochemical properties and digestion behaviour of chickpea protein, chickpea protein concentrate (CPC) was treated with electron beam irradiation (EBI) at doses of 5, 10, 15, and 20 kGy. After irradiation, protein solubility first increased at 10 kGy and 15 kGy, and then decreased at the higher dose of 20 kGy. This was supported by SDS-PAGE, where the intensity of major protein bands first increased and then decreased. Increased doses of EBI generally led to greater oxidative modification of proteins in CPC, indicated by reduced sulfhydryls and increased carbonyls. In addition, the protein structure was modified by EBI as shown by Fourier transform infrared spectroscopy analysis, where α-helix generally decreased, and β-sheet increased. Although the protein digestibility was not significantly affected by EBI, the peptidomic analysis of the digests revealed significant differences among CPC irradiated with varying doses. A total of 337 peptides were identified from CPC irradiated with 0 kGy, 10 kGy, and 20 kGy, with 18 overlapping peptides and 60, 29, and 40 peptides specific to the groups of 0, 10, and 20 kGy respectively. Theoretical calculation showed that the distribution of peptide length, hydrophobicity, net charge, and C-terminal residues were affected by irradiation. The 2, 2′-azino-bis (3-ethylbenzothiazoline-6-sulfonic acid) (ABTS) radical scavenging activity showed a marginal decrease with an increasing dose of irradiation. In conclusion, EBI led to oxidative modification and structural changes in chickpea protein, which subsequently affected the physicochemical properties of peptides obtained from in-vitro digestion of CPC, despite similar digestibility.

## 1. Introduction

Plant proteins are generally more readily available, less costly, and more environmentally friendly compared to animal proteins [1]. Chickpea (*Cicer arietinum* L.) is one of the most abundant legumes grown around the world, and chickpeas are also very nutritious, containing a variety of beneficial compounds including carbohydrates, protein, unsaturated fatty acids, minerals, vitamins, dietary fibre, and a range of isoflavones [2]. Chickpea has a high protein content, around 13–31% [3,4]. In addition, chickpea proteins have high bioavailability, a balanced amino acid composition, and good digestive properties [5]. As such, chickpea has had extensive research interest [6,7]. In recent years, plant proteins-derived digestion products have been widely studied due to the potential health implications of dietary peptides [8]. Chandrasekaran and Gonzalez [9] showed that chickpea protein hydrolysates produced from germinated chickpeas can be used to generate models to optimize the production of anti-diabetic peptides, and indicated that chickpea peptides can be used as commercial functional food ingredients. Acevedo and Gonzalezde [10] showed that chickpea hydrolysates contributed to the prevention and management of type 2 diabetes, and the chickpea protein digests of pepsin and trypsin showed good dipeptidyl peptidase IV inhibition potential.

Food irradiation is the process of exposing food to ionising and non-ionising radiation to destroy bacteria, viruses, or insects that may be present in the food [11]. It is considered to be an economical, safe, and environmentally friendly technology [12,13]. Electron beam irradiation (EBI), which consists of high-energy electrons and does not involve the use of radioactive material, can inactivate microorganisms by damaging critical components of cells [14]. In recent years, EBI has been widely used in food preservation [15,16]. The free radicals generated during EBI processing can also affect the critical components of the food matrix. It has been demonstrated that EBI can modify protein structure and therefore alter the protein functionality. Zhang et al. [17] showed that EBI reduced the content of α-helix and β-sheet in the secondary structure of the okara protein, increased the surface hydrophobicity and solubility, and improved the emulsifying properties of the okara protein. Liu et al. [18] showed that the S-S bond was broken into an SH bond after EBI, and the α-helix was transformed into β-sheet and unfolded due to the cleavage of covalent bonds, which exposed the buried hydrophobic amino acids of egg white protein; then, the antioxidant activity of egg white protein was improved.

EBI has been used to induce mutation with the hope of finding an improved cultivar of chickpea [19]. However, that was aimed at living plants. The technology can also be applied to final seeds, flours, protein concentrates, etc. In this regard, the effects of EBI on the functional and nutritional value of chickpea proteins are generally lacking. Zhang et al. [20] claimed that EBI was capable of improving food proteins mainly in four aspects: (1) the functional improvement of proteins; (2) promoting crude protein digestibility; (3) enhancing the biological activities of proteins; (4) facilitating the proteolytic effect via the modification of the protein structure. Therefore, we hypothesized that EBI treatment, initially used to prolong the shelf-life of food ingredients, would cause physicochemical changes in chickpea proteins and subsequently affect its nutritional value due to the side effect of irradiation-induced oxidation. In order to test this hypothesis, chickpea protein concentrates (CPC) were subjected to EBI at various doses. Protein solubility, oxidative modifications, advanced structures, and the peptidomic profile of the digested products were characterized.

## 2. Results

### 2.1. The Effect of EBI on the Amino Acid Residues of CPC

Amino acid composition can be used to evaluate protein modification [21]. Generally, the amino acid composition of CPC was not affected by EBI (Table 1). There were only minor changes in serine, arginine, tyrosine, phenylalanine, isoleucine, and lysine after EBI. Those amino acids appeared to be increased in groups with higher doses of irradiation.

**Figure 1:**
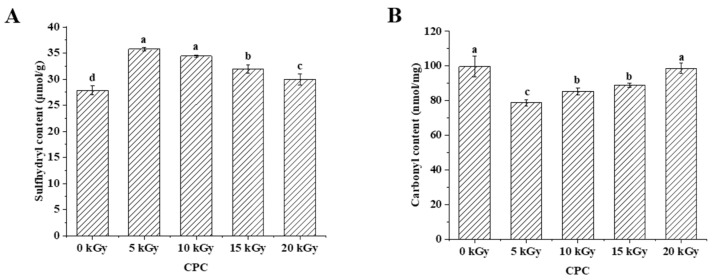
Effect of EBI on sulfhydryl content (**A**) and carbonyl content (**B**) of CPC. Different letters (a, b, c, d) indicate significant differences (*p* < 0.05).

The sulfhydryl group is susceptible to oxidative modification and it has an important contribution to protein functionality [22]. Oxidation of the sulfhydryl group may therefore affect the protein functionality. Results showed that the total sulfhydryl content of CPC was higher in irradiated groups than in the unirradiated group (Figure 1A). With an increased irradiation dose, sulfhydryl content decreased. Protein carbonyl content has been widely used as an indicator to evaluate protein oxidation [23]. The carbonyl content increased with an increasing irradiation dose, but the control group was higher than groups of 5–15 kGy (Figure 1B).

### 2.2. The Effect of EBI on the Physicochemical Properties of Proteins in CPC

**Figure 2:**
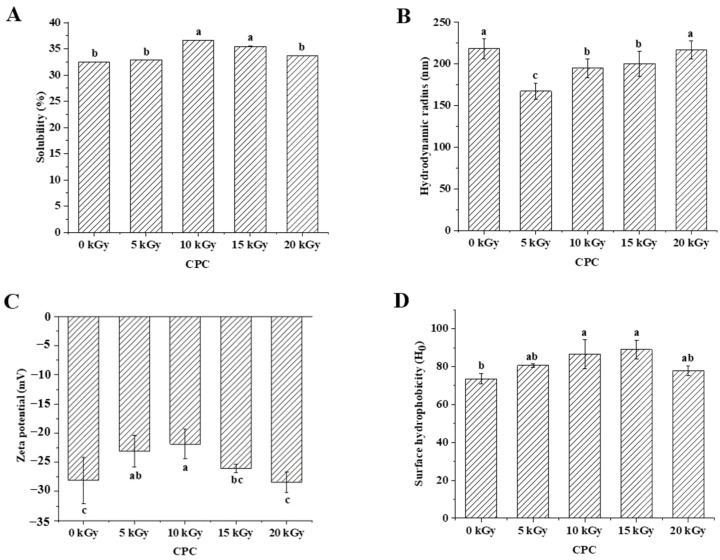
Effect of EBI on the solubility (**A**), hydrodynamic diameter (**B**), zeta potential (**C**), and surface hydrophobicity (**D**) of CPC. Different letters (a, b, c) indicate significant differences (*p* < 0.05).

**Figure 3:**
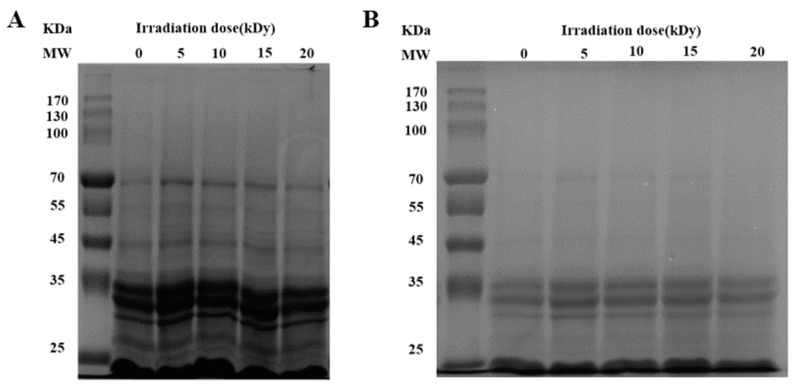
SDS-PAGE profiles of CPC under reducing (+DTT) conditions. MW: molecular weight marker (kDa). (The amount of protein loaded in (**A**,**B**) graphs were 32 μg and 25.6 μg, respectively).

Solubility is an important physicochemical property of proteins, reflecting the interactions between proteins and between proteins and water, and can influence the functional properties of proteins [24]. Figure 2A showed that the solubility of CPC increased and then decreased with an increasing irradiation dose. The solubility increased significantly at a dose of 10 kGy compared to the unirradiated samples. There was no further increase when the dose increased to 15 kGy. When the dose reached 20 kGy, the solubility decreased to a similar level of unirradiated. The solubilized proteins were subjected to SDS polyacrylamide gel electrophoresis analysis, and the results showed that the intensity of major protein bands was greater in CPC irradiated with 5, 10, and 15 kGy (Figure 3).

**Figure 4:**
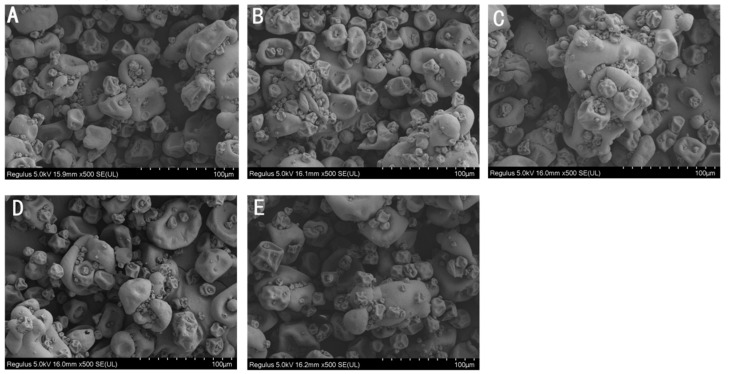
Scanning electron microscopy of CPC treated by irradiation at the following irradiation doses: (**A**)-0 kGy, (**B**)-5 kGy, (**C**)-10 kGy, (**D**)-15 kGy, and (**E**)-20 kGy, respectively.

Protein solubility is affected by its physiochemical properties, including particle size [25], net charge [26], surface hydrophobicity [27], etc. The hydrodynamic diameter of irradiated CPC was generally less than unirradiated CPC (Figure 2B). Increased irradiation generally led to increased hydrodynamic diameter. The SEM image of CPC powder also showed a higher proportion of large particles in irradiated groups (Figure 4).

In general, the zeta potential of CPC was between −20 and −30 mV, and irradiation at lower doses (5 and 10 kGy) led to a lower absolute value of zeta potential (Figure 2C). It was observed that irradiation at 10 and 15 kGy led to a higher surface hydrophobicity (Figure 2D).

Fourier transform infrared spectroscopy (FTIR) was used to study the effect of EBI on the secondary structure of chickpea protein. As shown in Table 2, the secondary structure of CPC is dominated by the β-sheet structure. With increasing irradiation doses, the content of α-helix generally decreased while β-sheet increased.

### 2.3. The Effect of EBI on the Peptidomics of CPC

The digestibility of protein is closely related to its nutritional quality. The in vitro digestibility of CPC of unirradiated and irradiated at doses of 5 kGy, 10 kGy, 15 kGy, and 20 kGy were 94.35%, 94.25%, 93.96%, 94.28%, and 93.84%, respectively, all exceeding 93% but without significant differences among groups.

**Figure 5:**
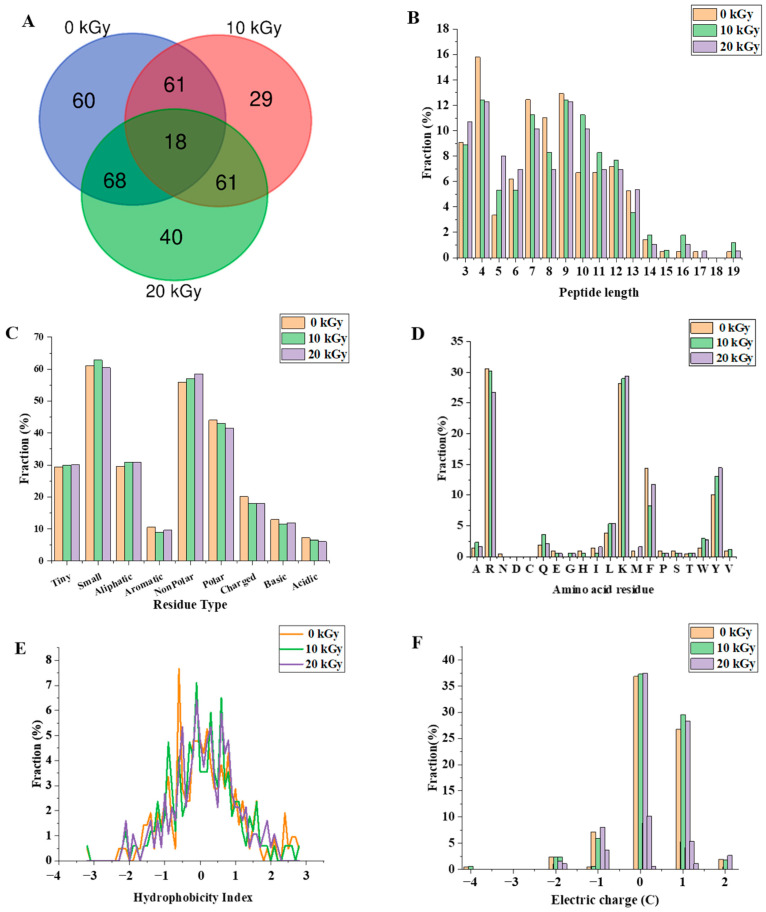
The effect of EBI on the characteristics of the gastrointestinal digestion products of CPC. (**A**) number of peptides released, (**B**) percentage of lengths of identified peptides, (**C**) percentage of residue group types, (**D**) distribution of C-terminal amino acid residues of peptides, (**E**) distribution of peptide hydrophobicity index (Kyte−Doolittle scale) and (**F**) peptide charge distribution.

**Figure 6:**
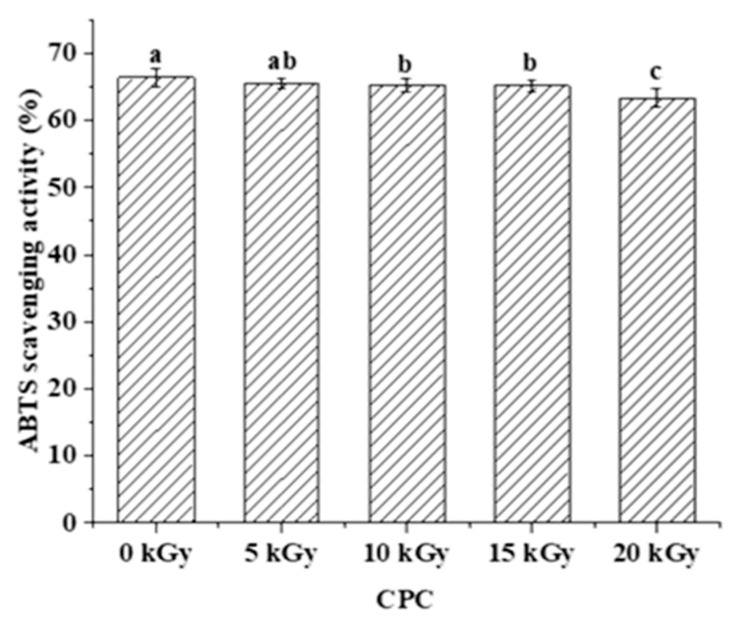
The effect of EBI on the ABTS radical scavenging activity of the gastrointestinal digestion products of CPC. Different letters (a, b, c) indicate significant differences (*p* < 0.05).

The digested CPC (0, 10, and 20 kGy) was further analysed through peptidomics. A total of 337 peptides were identified from the gastrointestinal digestion products of CPC treated with 0 kGy, 10 kGy, and 20 kGy irradiation doses (Figure 5A), with 18 overlapping peptides and 60, 29, and 40 peptides specific to 0, 10, and 20 kGy irradiation doses, respectively. A full list of those peptides can be found in the Supplementary File. The majority of peptides identified had a length of 3–13 residues (Figure 5B), with only about 2% having more than 14 residues. Irradiation generally led to a higher proportion of larger peptides. In terms of the amino acid composition of the peptides, more non-polar amino acid residues were found in irradiated groups (Figure 5C). When the C-terminal was considered alone, it was found that the two major residues were lysine and arginine, and irradiation appeared to increase the frequency of lysine while decreasing the frequency of arginine in the C-terminal of identified peptides (Figure 5D). The theoretical hydrophobicity and net charge of the peptides were also estimated (Figure 5E,F). The overall mean value of hydrophobicity was estimated using the Kyte-Doolittle scale, where scores below 0 indicate hydrophilicity, and values above 0 indicate hydrophobicity [28,29]. Overall, there were slightly more hydrophobic than hydrophilic peptides. The net charges of the three sample peptides were mainly 0 C and 1 C. The generated peptides may have various potential biological activity. The ABTS radical scavenging test showed that the antioxidant capacity of the digested products decreased slightly with increased irradiation (Figure 6).

## 3. Discussion

### 3.1. Oxidative Modification of Chickpea Protein

EBI employs high-energy electron beams to damage critical components of microorganisms, and thus extends the shelf-life of irradiated foods. However, when the high-energy electrons encounter water molecules, radiolysis of water occurs and this generates highly reactive free radicals (e.g., hydroxyl radicals, H^+^ ions, hydrated protons, hydrogen peroxide) [30]. The free radicals generated in EBI can lead to oxidative modifications of food components. In this study, it was found that increasing irradiation from 5 kGy to 20 kGy generally led to enhanced protein oxidation in CPC, indicated by the decreased sulfhydryl group (Figure 1A) and increased carbonyl group (Figure 1B). Similarly, increased irradiation doses promoted protein oxidation in various foods [17,31,32]. In the unirradiated CPC, protein oxidation seems to be more severe than irradiated ones, especially at lower doses of 5–15 kGy. This may be attributed to the release of bound phenolic compounds and destruction of the activity of endogenous lipoxygenase by EBI, as the decomposition of the phenolic compounds such as genistin and daidzin would lead to the formation of aglycone forms with better antioxidant potential compared to the bound forms [33]. Therefore, irradiation at lower doses resulted in the initial decrease in protein oxidation. With increased levels of irradiation, the excessive production of harmful free radicals can offset the beneficial effects observed at lower doses, resulting in increased protein oxidation. Loss of parent amino acids has been suggested as another indicator for the quantification of protein oxidation in biological systems [34]. In this study, the amino acid composition of CPC in different groups did not change much (Table 1). This suggested that EBI did not lead to severe protein oxidation in CPC. According to Lund et al. [35], amino acid analyses are most appropriate for the evaluation of large losses of amino acid residues. It is often difficult to detect small losses due to poor method sensitivity. Moreover, the hydrolysis during sample preparation may have destroyed some highly oxidizable amino acid residues, such as Met, Trp, and Cys.

### 3.2. Physicochemical Properties of Irradiated Chickpea Protein

Protein oxidation is known to affect the physicochemical properties and eventually the functionality of food proteins [36]. Formation of protein aggregation is one of the general consequences of protein oxidation. In the dry state, SEM images showed a higher proportion of large particles in irradiated CPC than unirradiated ones (Figure 4). This was supported by particle size measurement in the solubilized state (Figure 2B). Liu et al. [18] also found that the particle size of EBI-treated egg white proteins was significantly larger than that of untreated egg white protein, and they attributed the protein aggregation to an increased surface hydrophobicity, as EBI exposed the buried hydrophobic amino acids. The hydrodynamic radius of the CPC solution increased with an increased irradiation dose and reached a value around 220 nm at 20 kGy. The large particle size at 20 kGy may have caused a reduction in solubility, which was also observed in SDS-PAGE; the intensity of major protein bands in CPC irradiated with 20 kGy was less than that of 5, 10, or 15 kGy. Protein aggregates may be formed through covalent cross-linking or non-covalent interaction such as hydrophobic forces. In this study, no evidence of protein cross-linking was observed, as the SDS-PAGE did not show new bands in irradiated CPC. Hydrophobic interaction often leads to protein aggregation. It was observed that the surface hydrophobicity of CPC was greater at 10 and 15 kGy compared to unirradiated CPC (Figure 2D). The increase was likely due to protein structural changes, which may expose previously buried hydrophobic amino acid residues to the surface. FTIR analysis revealed that with increasing irradiation doses, the content of α-helix generally decreased while β-sheet increased (Table 2). This secondary structural change agreed with the general increase of surface hydrophobicity, as Kato and Nakai [37] found that the surface hydrophobicity of the protein was negatively correlated with the *α*-helical content. Similar structural changes were reported in EBI-treated myofibrillar proteins [32]. At 20 kGy, surface hydrophobicity showed a decreasing trend, likely due to extensive protein aggregations. It was found that EBI up to a dose of 15 kGy increased the solubility of CPC, followed by a decrease (Figure 2A). A similar trend was reported in EBI-treated okara protein, where the solubility increased at lower doses and decreased at higher doses. The altered solubility was attributed to protein unfolding and deamination during irradiation [38]. In this study, the increased hydrophobicity (Figure 2D) and decreased zeta-potential (Figure 2C) was expected to lower the solubility of CPC irradiated with 10 or 15 kGy; some other factors may have contributed to the observed solubility increase. However, this requires further investigation.

### 3.3. Characterization of the Peptides of In-Vitro Digested Chickpea protein

Amino acid side chain modification and protein structural changes are known to affect protein digestibility. Generally, low degrees of oxidation were found to enhance digestion by exposure of susceptible sites of the proteins to digestive enzymes, while high degrees of oxidation decrease protein susceptibility to digestive enzymes because of extensive side-chain modifications or protein aggregation [39]. Similar oxidation–functionality relationships have been observed in proteins from different origins [40]. In this study, in vitro digestibility was not significantly affected by EBI even though there was significant protein oxidation and structural changes. Despite similar digestibility, peptidomic analysis revealed significant differences among the three groups of CPCs. Peptides from these groups differed in their composition and physicochemical properties (Figure 5). Irradiation generated new peptides, while it also led to a diminishment of some other peptides which can only be found in unirradiated CPC. Increasing the dose from 10 kGy to 20 kGy led to an even greater number of new peptides. As there were differently abundant peptides (For full list, see Supplementary File) in each group, it was expected that the physicochemical properties would vary. This expectation was supported by a theoretical analysis of the peptides which showed that the distribution of peptide length, hydrophobicity, net charge, and C-terminal residues were affected by irradiation. The theoretical analysis of the peptide mixture was also performed by Boachie et al. [28] who investigated how the interaction with tannic acid affected the peptides of digested lentil proteins. The digestion of irradiated CPC generally led to a higher proportion of large peptides, possibly due to the chemical modification and structural changes. According to Xiong et al. [40], oxidative modification can change the proteolytic sites; this was supported by our findings that the frequency of the top four kinds (R, K, Y, F) of C-terminal amino acid residues were affected by irradiation. Of those four residues, arginine and lysine are the major cleavage sites of trypsin, while tyrosine and phenylalanine correspond to the major cleavage sites of pepsin. Trypsin, being a serine endopeptidase that hydrolyzes specifically proteins at the carboxy side of the basic amino acids (arginine and lysine), can be disrupted by the oxidation of NH_2_ groups of protein substrate into carbonyls [41]. Both chickpea protein [42] and its hydrolysate [5] have been demonstrated to possess antioxidant properties. In this study, the antioxidant capacity of the digested products was negatively affected by irradiation, but with only a marginal decrease (Figure 6). However, the antioxidant capacity was only assessed as ABTS radical scavenging activity, and the ABTS assay has its own limitation despite its wide application [43]. Other assays are encouraged to be performed, such as a DPPH (2,2′-diphenylpicryl hydrazyl free radical) assay, ORAC—oxygen radical absorbance capacity, etc. In addition to antioxidant activity, the digested products may have a range of biological activity such as antimicrobial activity, ACE inhibitory activity, etc. The peptides identified in this study can be compared to a database of biological active peptides, thereby serving as an initial screen step for further studies.

## 4. Materials and Methods

### 4.1. Materials and Electron Beam Irradiation

Chickpea protein concentrate, CPC (protein: 64.9%, moisture: 4.46%, ash: 3.90%), was purchased from Shandong Jianyuan Biological Engineering Co. Pepsin (enzyme activity >3000 units/mg) was purchased from Shanghai Maclean Biochemical Technology Co. Trypsin (enzyme activity >2500 units/mg) was purchased from Shanghai Yuanye Biotechnology Co. All other chemicals were of analytical grade and purchased from Sinopharm Chemical Reagent Co., Ltd. (Shanghai, China). For the electron beam irradiation, CPC in powder form were exposed to different doses (0, 5, 10, 15, and 20 kGy) of high-energy electron beam (produced from a 10 MeV, 25 kW electron-beam accelerator) at a dosage rate of 2 kGy/h. Different doses were achieved through exposures of different times. The levels of irradiation were chosen mainly based on the rules that an overall average irradiation dose of 10 kGy introduces no special nutritional or microbiological problems in foods (Codex General Standard for Irradiated Foods No. 106–1983).

### 4.2. Amino Acid Content

CPC (150.0 mg) was weighed into a hydrolysis tube, 8 mL of 6 M HCl was added, and nitrogen was used to remove the air in the hydrolysis tube. The tubes were then placed in an oven at 120 °C and hydrolyzed for 22 h. The hydrolysates were then transferred to a volumetric flask, neutralized by adding 4.8 mL of 10 M NaOH, and the volume was adjusted to 25 mL with deionized water. The suspensions were centrifugated at 5000 r/min for 5 min at room temperature (Model TGL-16gR, Shanghai Anting Scientific Instrument Factory, Shanghai, China), and the supernatant was subject to amino acid analysis according to the method of Jiang et al. [44].

### 4.3. Oxidative Modification of Amino Acids

#### 4.3.1. Sulfhydryl Content

Total sulfhydryl content was determined using Ellman’s reagent (DTNB) according to the method of Cheng et al. [45]. The absorbance was measured at 412 nm (Model UV1601, Riley Analytical Instruments, Beijing, China).

(1) Total sulfhydryl content(μmol/g)=73.53 × A412×DC

*A*_412_, the absorbance at 412 nm; D, dilution factor; and C, protein concentration (mg protein/mL).

#### 4.3.2. Carbonyl Content

The carbonyl content was measured using 2,4-dinitrophenyl hydra-zine (DNPH). CPC (0.03 g) were suspended with 3 mL 0.1 M Tris-HCl (pH 8.0, 5% sodium dodecyl sulphate *w*/*v*). The suspension was heated at 80 °C for 30 min and then cooled to room temperature. Then, the suspension was subject to determination of carbonyl content according to the method of Yu et al. [46]. The carbonyl content was calculated according to the equation:

(2) $$\mathrm{Carbonyl}\;\mathrm{content}\left(\mathrm{nmol}/\mathrm{mg}\right)=\frac{A_{370}}{22000\times \left(A_{280}-A_{370}\times 0.43\right)}$$

### 4.4. Solubility of CPC

The protein concentration of CPC was determined using the biuret reagent according to the method of Ji et al. [47], with some modifications. A 1% (*w*/*v*) CPC suspension was first centrifuged (8000 r/min, 4 °C, 10 min), and then 1 mL of the supernatant was mixed with 4 mL of biuret reagent. After a water bath at 37 °C for 20 min, the absorbance at 540 nm was measured to obtain the protein concentration. Protein solubility was defined as protein content in the supernatant divided by total protein in the suspension.

### 4.5. Particle Size and Zeta Potential of CPC

A 1% (*w*/*v*) CPC suspension was first centrifuged (8000 r/min, 4 °C, 10 min). The protein concentration of supernatant was determined using the biuret reagent, and then the protein concentration of the supernatant was adjusted to 1 mg/mL. The particle size and zeta potential of the CPC were then measured at 25 °C using a particle size meter (Model Litesizer 500, Anton Paar Trading Ltd., Houston, TX, USA). The parameters of the test were as follows: equilibration time of 60 s, and the test temperature of 25 °C. The refractive index was set to 1.46 and the absorption coefficient to 0.01.

### 4.6. Surface Hydrophobicity

The surface hydrophobicity of CPC was determined using the 8-Anilino-1-naphthalenesulfonic Acid (ANS) fluorescent probe method, slightly modified from Gulseren et al. [48]. CPC (1 mg/mL) was first dispersed in 0.01 M phosphate buffer (Na_2_HPO_4_-NaH_2_PO_4_, pH 7.0). The protein suspension was then centrifuged (8000 r/min, 4 °C, 15 min), and the protein concentration of the supernatant was determined using the biuret reagent as mentioned above. Next, the supernatant was diluted with phosphate buffers to 0.5, 0.1, 0.025, 0.005, and 0.001 mg/mL. ANS was dissolved in 0.01 M (pH 7.0) phosphate buffer and 40 μL of ANS solution was added to 4 mL protein solution. Absorbance was then measured using a fluorescence spectrophotometer (Model Cary Eclipse, Agilent Technologies Ltd., Santa Clara, CA, USA) at an excitation wavelength of 280 nm and an emission wavelength of 355 nm. The H_0_ index was calculated as the initial slope of the fluorescence intensity versus protein concentration by linear regression analysis.

### 4.7. Fourier Transform Infrared Spectroscopy (FTIR) Analysis

CPC was mixed with KBr (1:200), ground, and then a total of 200 mg of the mixture powder was pressed into a narrow slice. According to the method of Wang et al. [49], the Fourier transform infrared (FTIR) spectra were recorded by a FTIR spectrometer (Model Nicolet iS10, Thermo Fisher Scientific, Waltham, MA, USA). The wavelength range was measured from 4000 to 400 cm^−1^. The obtained FTIR spectra were further processed using Omnic V8.1 (Version V8.1, Thermo Fisher Scientific, USA) and PeakFit 4.12 (Version 4.12, Systat Software, San Jose, CA, USA). Baseline correction, deconvolution, and second-order derivatives were performed on the original data at a fraction of 1600–1700 cm^−1^. The following ranges were assigned to each secondary structure: β-sheet (1615–1,637 cm^−1^, 1682–1700 cm^−1^), random coil (1637–1645 cm^−1^), α-helix (1646–1664 cm^−1^), and β-turn (1664–1681 cm^−1^). The percentages of each secondary structure were calculated to present the relative content of secondary structures in the CPC.

### 4.8. SDS Polyacrylamide Gel Electrophoresis (SDS-PAGE)

Electrophoresis was carried out with 12% separating gel and 4% stacking gel according to the method of Wang et al. [50] under reduced (+DTT) conditions. The CPC solution (80 μL, 4 mg/mL) was mixed with 20 μL 5× sample buffer. Then, the mixture was heated under 95 °C for 10 min and cooled to room temperature. Later, the mixture (10 μL, 8 μL) was loaded into the gel. Electrophoresis was first carried out at a voltage of 80 V for 30 min and then 120 V for 110 min. Next, Coomassie brilliant blue R-250 was used to stain the gel for 30 min. After destaining overnight, the gel was scanned with a multifunctional gel image analysis system (Model Tanon MINI Space, Shanghai Tianneng Life Science Co., Ltd., Shanghai, China).

### 4.9. Scanning Electron Microscopy (SEM)

Chickpea protein powder was freeze-dried (Model FD-1A-50, Boyikang (Beijing) Instrument Co., Ltd., Beijing, China), and protein powder after freeze-drying was subject to scanning electron microscope observation according to the method of Zhang et al. [51].

### 4.10. Intrinsic Fluorescence Spectroscopy (IFS)

The method of Jiang et al. [52] was used to determine intrinsic fluorescence spectroscopy, and the method was slightly modified. CPC (1 mg/mL) was first dispersed in 0.01 M phosphate buffer (Na_2_HPO_4_-NaH_2_PO_4_, pH 7.0). The protein suspension was then centrifuged (8000 r/min, 4 °C, 15 min), and the protein concentration of supernatant was determined with the biuret reagent as mentioned above. Next, the supernatant was diluted using phosphate buffers to 0.5 mg/mL. The fluorescence intensity of the samples was performed with a fluorescence spectrophotometer (Model Cary Eclipse, Agilent Technologies Ltd., USA) at room temperature. Intrinsic spectra were recorded between 300 and 450 nm with an excitation wavelength of 280 nm (slit = 2.5 nm) at 10 nm s^−1^ of scanning speed.

### 4.11. In Vitro Digestion of CPC

#### 4.11.1. In Vitro Digestibility

The method of Wen et al. [53] was referenced with slight modifications. CPC (0.32 g) was added to deionized water (16 mL), and the pH of the suspension was adjusted to 2.0 ± 0.1 with 1 M HCl. Pepsin premix (0.8 mg/mL, 4 mL) was added to each sample. The mixture was shaken uniformly at 37 °C for 1 h. The pH of the suspension was quickly adjusted to 7.5 ± 0.1 with 1 M NaOH. The mixture was added to 4 mL of trypsin premix (0.24 mg/mL), and the mixture was shaken uniformly at 37 °C for 2 h. The enzymatic reaction was terminated by heating it at 95 °C for 5 min, and 4 mL of the mixture was removed as the gastrointestinal digested products. The gastrointestinal digested products were added to 12 mL of anhydrous ethanol at 4 °C and left to stand for 12 h. The product was centrifuged (8000 r/min, 15 min, 4 °C). The precipitate after separation is used to determine the protein content after digestion, and the supernatant is used for peptidomic identification. The digestion of CPC samples was repeated another two times.

The in vitro digestibility of proteins is determined as follows:

(3) $$\mathrm{Protein}\;\mathrm{digestibility}\;\mathrm{in}\;\mathrm{vitro}\left(\%\right)=\frac{M_{1}-M_{2}}{M_{1}}\times 100$$

M_1_, protein content of the sample before digestion; M_2_, the protein content of the sample in the precipitate after digestion.

#### 4.11.2. Peptidomic Analysis

Unirradiated and irradiated samples (doses of 10 kGy and 20 kGy) were taken from the alcohol-soluble supernatant of the gastrointestinal digest of CPC (described in 4.11.1). The supernatant was freeze-dried (Model FD-1A-50, Boyikang (Beijing) Instrument Co., Ltd., China). The obtained powder of peptides was re-dissolved in 0.1% formic acid and analysed by Q-Exactive Plus coupled to an EASY-nano LC 1200 system (Thermo Fisher Scientific, MA, USA). A 1 μL peptide sample was loaded onto a 25 cm analytical column (75 μm inner diameter, 1.9 μm resin) and separated with a 60 min gradient starting at 2% (80% acetonitrile with 0.1% formic acid) followed by a stepwise increase to 35% at 47 min, then 100% after 1 min, and remained there for 12 min. The flow rate was maintained at 300 nL/min, and the column temperature was set at 40 °C.

The mass spectrometer was run under data dependent acquisition (DDA) mode. The survey of full scan MS spectra (*m*/*z* 200–2000) was acquired in the Orbitrap with a resolution of 70,000.

Tandem mass spectra were processed by PEAKS Studio version 10.6 (Bioinformatics Solutions Inc., Waterloo, ON, Canada). The database was Cicer arietinum (24,812 entries) downloaded from uniprot. Pepsin and trypsin were the digestion enzymes and the digest type was semi-specific. PEAKS DB was searched with a fragment ion mass tolerance of 0.02 Da and a parent ion tolerance of 10 ppm. The max missed cleavage was set to two. Oxidation on methionine, and deamidation on asparagine and glutamine were specified as the variable modifications. The peptides with 1% FDR and the proteins with at least 1 unique peptide were filtered. “Match between runs” was enabled with default settings. Label-free quantification was also performed using Peaks Studio. A relative abundance of peptide features (precursor peak area) was detected in multiple samples. Feature detection is performed separately on each sample, and then the features of the same peptide from different samples are reliably aligned together using a high-performance retention time alignment algorithm. Normalization was performed on the total ion current (TIC) of the samples and normalized abundance was calculated from the raw abundance divided by the normalization factor.

A Venn diagram depicting the degree of similarity of peptides in the gastrointestinal digestion products of the three groups was generated using online tools (https://bioinformatics.psb.ugent.be/webtools/Venn/ (accessed on 20 June 2023)). Peptide properties were obtained from the Peptides R package [54].

#### 4.11.3. ABTS Radical Scavenging Capacity

The 2, 2′-azino-bis (3-ethylbenzothiazoline-6-sulfonic acid) (ABTS) in the digestion products of CPC was determined according to the method of Xing et al. [55]. Results were expressed as ABTS radical scavenging activity, calculated using the following equation:

(4) $$Scavenging\;activity\left(\%\right)=\frac{A_{734\;\left(blank\right)}-A_{734\;\left(sample\right)}}{A_{734\;\left(blank\right)}}\times 100$$

*A*_734 (*sample*)_, the absorbance of ABTS reacted with the sample at *A*_734_; *A*_734 (*blank*)_, the absorbance of ABTS reacted with distilled water at *A*_734_.

### 4.12. Statistical Analysis

All measurements were performed in triplicate. Results were expressed as mean ± standard deviation (SD). Data were analysed using IBM SPSS software (version 20.0, IBM, Chicago, IL, USA). One-way analysis of variance (ANOVA) was performed with *p* < 0.05 set as statistically significant.

## 5. Conclusions

In this study, it was found that electron beam irradiation induced significant protein oxidation and altered the structure and physicochemical properties of chickpea protein in a dose-dependent manner. Proteins were found to be aggregated, likely due to hydrophobic interaction rather than covalent protein cross-linking. The apparent digestibility of CPC at different doses was similar to that of unirradiated CPC; however, the peptide profile of the digests differed among CPC at 0, 10, and 20 kGy. Peptidomic analysis showed that the distribution of peptide length, hydrophobicity, net charge, and C-terminal residues were all affected by irradiation. Arginine, lysine, tyrosine, and phenylalanine were the major cleavage sites of trypsin or pepsin used in the in vitro digestion test; their frequency in the C-terminal were all affected, suggesting that irradiation altered the proteolytic behaviour of chickpea protein. The antioxidant capacity of the digests was negatively affected by irradiation, though it was only a minor effect. In this study, the reason for the increased solubility of CPC with an increased dose of irradiation, as well as the reduction of hydrodynamic radius in the supernatant of CPC at 5 kGy, require further exploration. This study provided theoretical guidance in the application of electron beam irradiation technology in chickpea protein concentrate.

## Figures and Tables

**Table 1:** Effect of EBI on the amino acid content of CPC.

Amino Acid Content g/100 g	0 kGy	5 kGy	10 kGy	15 kGy	20 kGy
asp	8.51 ± 0.09 ^a^	8.54 ± 0.07 ^a^	8.55 ± 0.12 ^a^	8.41 ± 0.04 ^a^	8.52 ± 0.13 ^a^
glu	13.37 ± 0.17 ^a^	13.46 ± 0.12 ^a^	13.50 ± 0.21 ^a^	13.27 ± 0.09 ^a^	13.44 ± 0.21 ^a^
ser	2.98 ± 0.08 ^b^	3.07 ± 0.07 ^ab^	3.15 ± 0.10 ^a^	3.12 ± 0.03 ^a^	3.16 ± 0.01 ^a^
his	1.69 ± 0.05 ^a^	1.73 ± 0.07 ^a^	1.68 ± 0.15 ^a^	1.68 ± 0.03 ^a^	1.65 ± 0.09 ^a^
gly	2.66 ± 0.03 ^a^	2.68 ± 0.03 ^a^	2.67 ± 0.03 ^a^	2.62 ± 0.04 ^a^	2.65 ± 0.04 ^a^
thr	2.1 ± 0.02 ^a^	2.12 ± 0.01 ^a^	2.13 ± 0.05 ^a^	2.1 ± 0.06 ^a^	2.15 ± 0.04 ^a^
arg	6.08 ± 0.09 ^b^	6.16 ± 0.04 ^ab^	6.21 ± 0.08 ^a^	6.11 ± 0.03 ^ab^	6.19 ± 0.05 ^ab^
ala	2.89 ± 0.02 ^a^	2.90 ± 0.03 ^a^	2.91 ± 0.04 ^a^	2.88 ± 0.02 ^a^	2.92 ± 0.04 ^a^
tyr	1.67 ± 0.06 ^b^	1.78 ± 0.03 ^a^	1.72 ± 0.03 ^ab^	1.71 ± 0.05 ^ab^	1.71 ± 0.03 ^ab^
cys-s	0.26 ± 0.01 ^a^	0.26 ± 0.03 ^a^	0.30 ± 0.03 ^a^	0.29 ± 0.03 ^a^	0.29 ± 0.01 ^a^
val	3.65 ± 0.04 ^a^	3.64 ± 0.04 ^a^	3.66 ± 0.05 ^a^	3.58 ± 0.05 ^a^	3.63 ± 0.05 ^a^
met	0.97 ± 0.05 ^a^	0.97 ± 0.09 ^a^	1.00 ± 0.07 ^a^	0.98 ± 0.05 ^a^	0.97 ± 0.03 ^a^
phe	4.31 ± 0.06 ^b^	4.35 ± 0.03 ^ab^	4.36 ± 0.06 ^ab^	4.29 ± 0.02 ^b^	4.41 ± 0.07 ^a^
ile	3.45 ± 0.03 ^ab^	3.47 ± 0.00 ^a^	3.46 ± 0.03 ^a^	3.39 ± 0.04 ^b^	3.44 ± 0.04 ^ab^
leu	5.48 ± 0.07 ^a^	5.53 ± 0.04 ^a^	5.55 ± 0.07 ^a^	5.47 ± 0.03 ^a^	5.54 ± 0.06 ^a^
lys	4.33 ± 0.03 ^ab^	4.39 ± 0.07 ^ab^	4.39 ± 0.02 ^ab^	4.32 ± 0.01 ^b^	4.41 ± 0.07 ^a^
pro	2.93 ± 0.38 ^a^	2.78 ± 0.13 ^a^	3.17 ± 0.31 ^a^	3.01 ± 0.25 ^a^	2.94 ± 0.11 ^a^

Different superscript letters in the same row indicate significant differences (*p* < 0.05).

**Table 2:** Effect of EBI on the percentage of the secondary structure of CPC analysed by FTIR.

	β-Sheet	Random Coils	α-Helix	β-Turn
0 kGy	51.69 ± 0.93 ^b^	2.95 ± 0.30 ^b^	21.69 ± 0.16 ^a^	23.69 ± 1.07 ^a^
5 kGy	56.96 ± 2.81 ^a^	2.76 ± 0.53 ^b^	21.20 ± 1.47 ^ab^	19.08 ± 0.82 ^b^
10 kGy	57.10 ± 0.79 ^a^	4.29 ± 0.50 ^a^	20.53 ± 0.12 ^ab^	18.08 ± 1.17 ^b^
15 kGy	57.15 ± 1.25 ^a^	2.07 ± 0.19 ^b^	18.41 ± 0.53 ^c^	22.38 ± 0.53 ^a^
20 kGy	54.32 ± 0.60 ^ab^	2.11 ± 0.89 ^b^	19.60 ± 0.22 ^bc^	23.98 ± 0.07 ^a^

Different superscript letters indicate significant differences within columns (*p* < 0.05).

## Data Availability

Data will be made available upon reasonable request.

## Supplementary Materials

The following supporting information can be downloaded at: https://www.mdpi.com/article/10.3390/molecules28166161/s1, Supplementary File: Differentially abundant peptides in digested chickpea protein subjected to irradiation.
